# Beyond EMT: Mesenchymal drift as an emerging driver of stromal-immune reprogramming in prostate cancer

**DOI:** 10.1016/j.isci.2026.116666

**Published:** 2026-07-16

**Authors:** Zijian Zhang, Feifan Liu, Shuze Liu, Hao Ning, Yang Sun, Fei Wu

**Affiliations:** 1 Department of Urology, Shandong Provincial Hospital Affiliated to Shandong First Medical University, Jinan, Shandong Province, P.R. China; 2 Department of Dermatology, Qilu Hospital Shandong University, Jinan, Shandong Province, P.R. China

**Keywords:** Cancer, Cell biology, Immunology, Microenvironment

## Abstract

Cellular plasticity within the tumor microenvironment (TME) extends far beyond classical epithelial-mesenchymal transition (EMT). Emerging evidence indicates that diverse non-epithelial cell populations, including macrophages, endothelial cells, pericytes, adipocytes, and fibroblasts, undergo a progressive and often partial reprogramming toward mesenchymal-like states during tumor progression. We conceptualize this broader phenomenon as mesenchymal drift (MD), a *trans*-lineage adaptive process characterized by erosion of lineage-specific identity, acquisition of extracellular matrix-remodeling capacity, enhanced migratory potential, and epigenetic stabilization of pro-fibrotic and immunosuppressive programs. In prostate cancer (PCa), MD provides a conceptual framework for interpreting stromal-immune remodeling across epithelial, endothelial, immune, adipose, and fibroblastic compartments. Macrophage-to-myofibroblast transition (MMT), endothelial-to-mesenchymal transition (EndoMT), pericyte-to-fibroblast transition (PFT), and adipocyte mesenchymal transition (AMT) collectively expand the pool of cancer-associated fibroblasts, promote matrix stiffening, induce vascular dysfunction, and reinforce immune evasion. These processes are proposed to be driven by overlapping signaling networks—including TGF-β/Smad, Wnt/β-catenin, Hippo/YAP-TAZ, PDGF, and inflammatory NF-κB/STAT3 pathways—and are stabilized by DNA methylation, histone modifications, and non-coding RNAs. Clinically, MD-associated transcriptional signatures correlate with aggressive phenotypes, metastasis, and therapy resistance across solid tumors, including PCa, highlighting their potential as prognostic biomarkers and therapeutic targets. Pharmacologic inhibition of key MD drivers, epigenetic reprogramming strategies, and combinatorial approaches with immunotherapy represent promising translational avenues. By integrating diverse mesenchymal transition processes under a unified conceptual framework, this review positions mesenchymal drift as a unifying axis of stromal-immune reprogramming in prostate cancer and underscores its significance for next-generation therapeutic strategies.

## Graphical abstract

**Figure:**
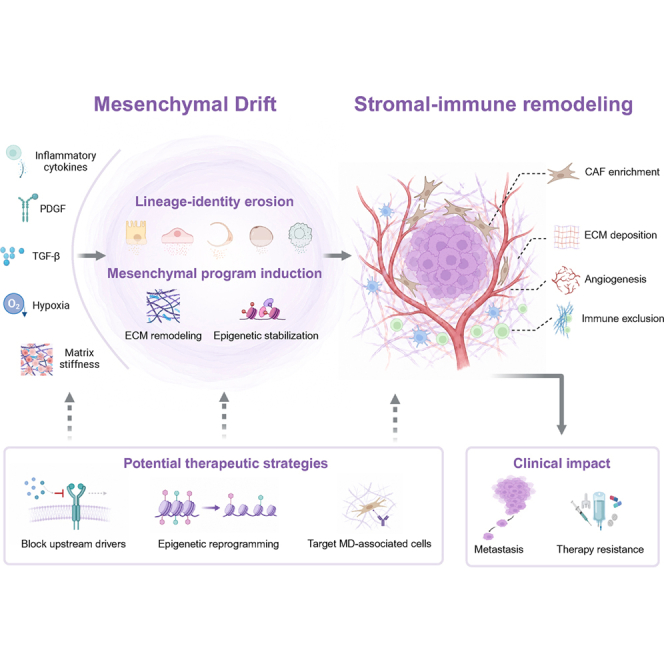

## Teaser

Health sciences; Medicine; Medical specialty; Internal medicine; Oncology

## Introduction

Prostate cancer (PCa) represents a major global health burden, being the second most common malignancy in men, with over 1.4 million new cases and nearly 394,000 deaths annually.^1^ Its incidence is projected to rise, potentially making it the most frequent cancer (excluding skin cancer) among men by 2025, accounting for 30% of new cases.^2^ Clinical outcomes vary markedly by disease stage: while localized PCa boasts a 5-year survival rate close to 100%, the prognosis for patients with locally advanced or metastatic disease declines significantly.^3^^,^^4^ This disparity highlights the pressing need to understand the drivers of tumor progression and therapy resistance. Recent studies have revealed that dynamic interactions within the tumor microenvironment (TME) play a critical role in mediating PCa initiation, progression, metastasis, and treatment failure. Cellular plasticity serves as a key mechanism enabling this adaptive capacity, which in cancer cells is classically observed through processes such as the epithelial-mesenchymal transition (EMT).^5^ Beyond the epithelium, accumulating evidence indicates that diverse non-epithelial components of the TME—including adipocytes, macrophages, and endothelial cells—can also undergo a marked phenotypic shift, acquiring mesenchymal-like characteristics in their gene expression, morphology, and function.^6^ This broader phenomenon has been termed “mesenchymal drift” (MD). MD describes an adaptive program wherein various cell types, under microenvironmental pressures, dynamically activate a suite of capabilities that confer mesenchymal cell-like motility and matrix-remodeling properties, thereby altering the TME.^6^ Illustrative examples include adipocyte mesenchymal transition (AMT) in breast cancer promoting a pro-tumorigenic niche,^7^ macrophage-to-myofibroblast transition (MMT) in oral squamous cell carcinoma (OSCC),^8^ and endothelial-mesenchymal transition (EndoMT) in gastric cancer fueling invasion and metastasis.^9^ This review aims to integrate current knowledge and provide a focused perspective on MD in the prostate stroma and immune landscape. We will delve into the molecular drivers that initiate MD, elaborate on its consequences for immune evasion and matrix remodeling, evaluate its clinical potential as a biomarker and therapeutic target, and outline future research directions to harness this understanding for improving PCa therapy.

## The concept and paradigm of MD

The MD concept represents a paradigm shift in our understanding of the plasticity of the TME. EMT is the process by which cells dynamically transform from an epithelial phenotype to a mesenchymal phenotype under the induction of specific signals.^10^ This process is characterized by loss of epithelial adhesion and acquisition of migratory and invasive properties.^11^^,^^12^ This process is initiated by signals such as TGF-β, and is primarily driven by the activation of transcription factors like ZEB1, which inhibits epithelial genes and initiates mesenchymal programs.^13^ During tumor progression, EMT mainly focuses on the ability of cancer cells themselves to acquire migration, stem cell characteristics, and therapeutic resistance. Meanwhile, EMT cells also shape the immunosuppressive microenvironment by upregulating PD-L1 and other mechanisms, promoting immune escape, thereby driving tumor metastasis and drug resistance.^14^

**Figure 1:**
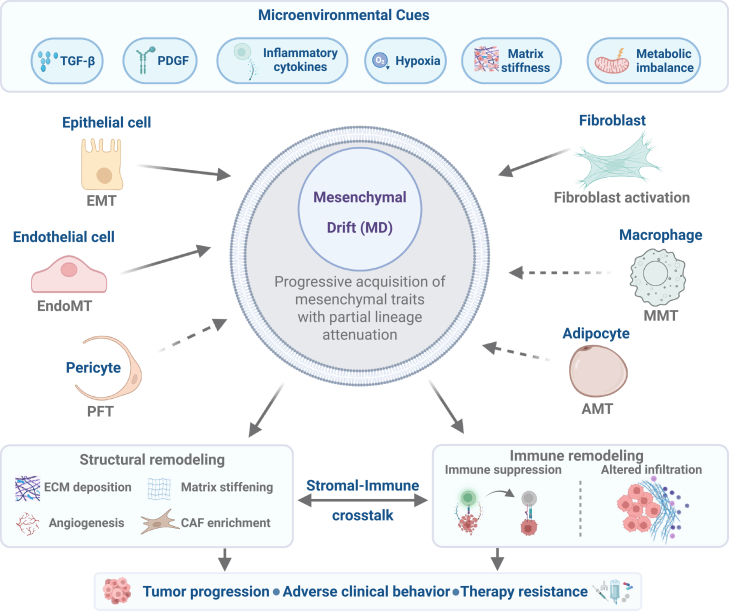
Mesenchymal drift as a conceptual framework for multi-cellular mesenchymal reprogramming in prostate cancer Schematic overview of mesenchymal drift (MD) within the prostate tumor microenvironment. Persistent microenvironmental cues—including TGF-β, PDGF, inflammatory cytokines, hypoxia, matrix stiffness, and metabolic imbalance—may promote mesenchymal reprogramming across multiple cellular compartments. Within this framework, epithelial cells (EMT), endothelial cells (EndoMT), pericytes (PFT), macrophages (MMT), adipocytes (AMT), and activated fibroblasts are illustrated as MD-related cellular programs that may contribute to tumor microenvironment remodeling under specific experimental or pathological conditions reported in the literature. Rather than implying complete lineage conversion, MD refers to the progressive acquisition of mesenchymal traits, accompanied by partial lineage attenuation, functional mesenchymal skewing, and context-dependent plasticity. These processes may contribute to structural remodeling, including extracellular matrix deposition, matrix stiffening, angiogenesis, and cancer-associated fibroblast enrichment, as well as immune remodeling characterized by immune suppression and altered immune infiltration. Structural and immune remodeling may further interact through stromal-immune crosstalk and are collectively associated with tumor progression, adverse clinical behavior, and therapy resistance. Solid arrows indicate relationships with relatively stronger support in prostate cancer or closely related contexts, whereas dashed arrows indicate more limited or context-dependent evidence. This framework is intended to integrate related mesenchymal transition phenomena under a shared conceptual logic and does not imply uniform occurrence, irreversibility, or complete transdifferentiation across all cell types or disease settings.

**Figure 2:**
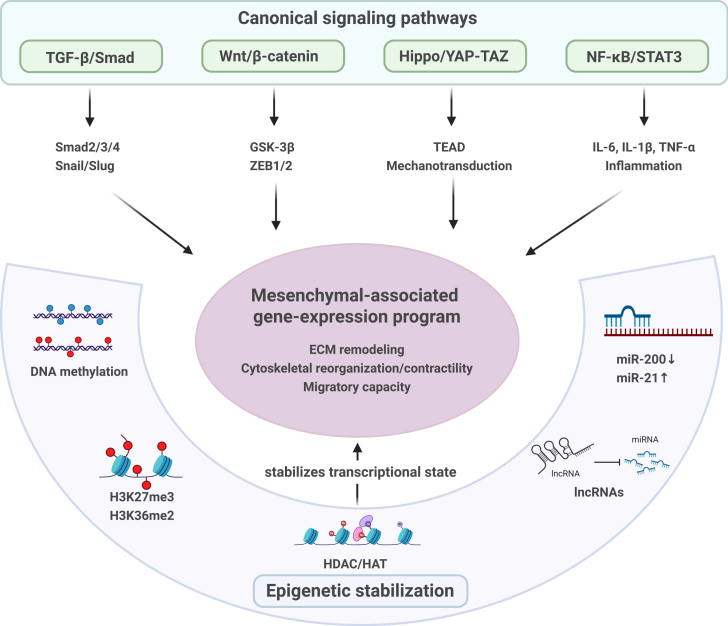
Integrated signaling and epigenetic networks implicated in mesenchymal drift Proposed signaling and epigenetic circuits that may contribute to mesenchymal reprogramming in prostate cancer. Canonical pathways—including TGF-β/Smad, Wnt/β-catenin, Hippo/YAP-TAZ, and inflammatory NF-κB/STAT3 signaling—have been independently implicated in related mesenchymal transition paradigms. These pathways act through pathway-linked regulatory mediators and processes and may functionally converge on mesenchymal-associated gene-expression programs, including extracellular matrix remodeling, cytoskeletal reorganization/contractility, and migratory capacity. Epigenetic mechanisms, including DNA methylation, histone modifications (e.g., H3K27me3, H3K36me2), and non-coding RNAs (e.g., miR-200 family, miR-21, lncRNAs), may stabilize or reinforce these transcriptional states. The diagram summarizes reported regulatory links across related transition paradigms and does not imply that all pathways operate simultaneously, uniformly, or with equal evidentiary support across all MD-related cell states.

**Figure 3:**
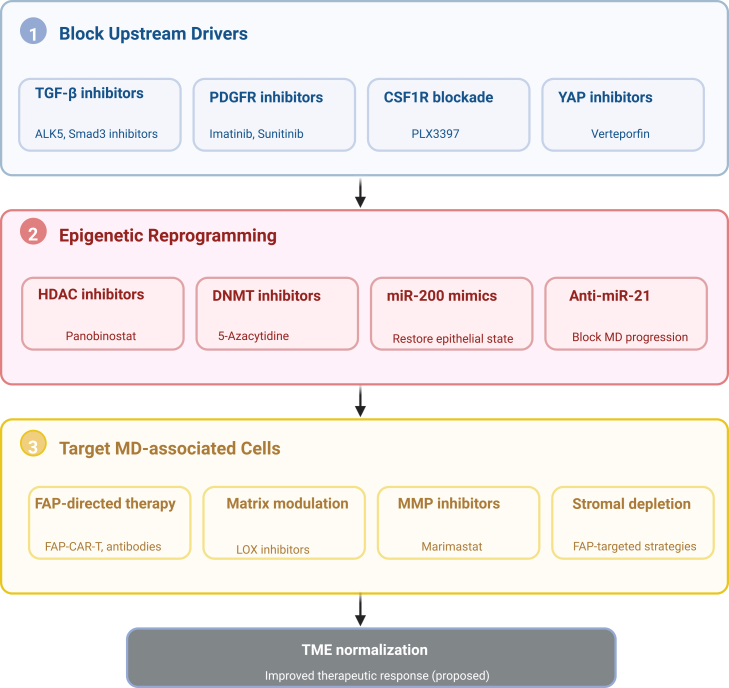
Potential therapeutic strategies to intercept mesenchymal drift and remodel the tumor microenvironment Conceptual overview of therapeutic strategies that may modulate mesenchymal drift (MD)-associated processes in prostate cancer. Rather than listing independent interventions for individual transition types, the figure maps potential therapeutic entry points within the shared MD framework, including: (1) inhibition of upstream signaling drivers (e.g., TGF-β, PDGFR, CSF1R, YAP/TAZ pathways); (2) modulation of epigenetic regulators that stabilize mesenchymal programs (e.g., HDAC or DNMT inhibitors, microRNA-based interventions); and (3) targeting mesenchymal-activated stromal populations (e.g., FAP-directed therapies or matrix-modifying strategies). These approaches are derived from studies across mesenchymal transition and tumor microenvironment research fields. Their specific efficacy in reversing mesenchymal drift in prostate cancer remains an area of ongoing investigation.

To better define the conceptual scope of MD, it is important to distinguish it from related but non-equivalent concepts (Box 1). EMT describes a relatively well-defined phenotypic plasticity program in epithelial cells characterized by loss of polarity and acquisition of migratory capacity.^10^ Transdifferentiation implies a direct, typically irreversible conversion of one differentiated cell type into another, usually requiring lineage-tracing evidence.^15^ By contrast, MD refers to a process in which cells undergo partial or complete cellular reprogramming, resulting in impaired original cellular characteristics and the acquisition of new functions or enhancement of existing stromal properties during tissue aging or disease progression (Figure 1). At the molecular level, the weakening of original cell identity markers, coupled with the upregulation of mesenchymal-related genes and proteins, leads to a functional shift toward fibrosis, matrix remodeling, migration, and other directions.^6^ MD represents a *trans*-lineage phenotypic convergence phenomenon that encompasses diverse, contextually regulated mesenchymal transition, including but not limited to EMT,^16^ EndoMT,^17^ MMT,^18^ and pericyte-to-fibroblast transition (PFT).^19^ As summarized in Figures 1, 2, and 3, we propose MD, not as a collection of parallel transition events, but as an integrative framework linking shared microenvironmental cues, convergent regulatory circuitry, and common functional consequences across stromal and immune compartments. In the TME, MD reprograms cell identity, forming a fibrotic matrix centered on cancer-associated fibroblasts (CAFs), promoting vascular remodeling and immunosuppression, thereby leading to accelerated tumor growth, increased metastasis rate, and treatment resistance.

Box 1 **What mesenchymal drift is and is not**

**MD is:**

A progressive, partial, and often reversible phenotypic skewing toward mesenchymal-like programs that can occur across multiple stromal and immune cell types under persistent microenvironmental pressures. It is characterized by attenuation of lineage-restricted identity together with induction of mesenchymal-associated markers and functions (for example, ECM remodeling, cytoskeletal reorganization, contractility, and increased motility), often manifesting as co-expression of residual lineage markers with newly acquired mesenchymal markers. MD is typically driven by sustained cues, such as TGF-β, inflammatory cytokines, hypoxia, metabolic stress, and mechanical forces, and can be epigenetically stabilized while remaining at least partly reversible in many contexts.

**MD is not:**

A complete, irreversible transdifferentiation into a fully specified mesenchymal lineage, nor a transient inflammatory activation or short-lived stress response. It should not be defined by single-marker expression or transcriptomic correlation alone without supporting evidence of lineage-identity erosion and functional remodeling. MD is not synonymous with EMT; rather, EMT can be considered an epithelial-restricted instance within a broader MD framework. Finally, MD should not be interpreted as a terminal differentiation endpoint, because many MD states are context-dependent and may revert if the driving cues are removed or therapeutically modulated.

Unlike classical descriptions of EMT, MD places greater emphasis on the gradual, incomplete nature of cellular reprogramming. At the molecular level, drifting cell populations often retain expression of some original epithelial markers while functionally acquiring definitive mesenchymal traits. This drift does not necessarily require a complete transformation into mesenchymal identity, but rather a pronounced transcriptional and functional skewing toward this state, which has been demonstrated in glioblastoma and anaplastic thyroid carcinoma.^20^^,^^21^ It emphasizes that cells exposed to specific microenvironment signals exhibit adaptive functional changes.^6^ Particularly compelling evidence comes from the study of circulating tumor cells. In lung cancer and breast cancer, many cells exhibit a hybrid epithelial/mesenchymal phenotype, which directly proves that tumor cells can stably exist in an intermediate state during metastasis without completing a complete mesenchymal transition.^22^^,^^23^

### Defining mesenchymal drift: Conceptual boundaries and core features

MD represents a broader and more plastic *trans*-lineage adaptive process occurring across multiple non-epithelial cell types within pathological microenvironments. MD is defined by four core features.

1. Erosion of lineage-restricted identity. Cells undergoing MD display partial attenuation of lineage-specific markers and functions without necessarily undergoing complete transdifferentiation.
2. Acquisition of mesenchymal functional traits. Rather than complete lineage conversion, drifting cells progressively upregulate genes associated with extracellular matrix remodeling, cytoskeletal reorganization, contractility, and migratory capacity (e.g., α-SMA, COL1A1, FN1, and VIM).
3. Microenvironment-driven and context-dependent plasticity. MD is induced by persistent pathological cues, such as TGF-β, inflammatory cytokines, mechanical stress, metabolic imbalance, and hypoxia and is dynamically regulated by stromal-immune crosstalk.
4. Epigenetic stabilization with retained reversibility. DNA methylation, histone modifications, and non-coding RNAs consolidate mesenchymal-skewed transcriptional programs, yet many MD states remain partially reversible, distinguishing them from terminal differentiation.

To avoid conflating transient marker fluctuations with genuine drift, we propose that the classification of a cellular process as MD requires concurrent evidence in the following three dimensions: (1) lineage-identity erosion—partial downregulation of lineage-specific transcription factors or surface markers relative to the native state; (2) mesenchymal program induction—upregulation of ECM remodeling, cytoskeletal, or contractility genes (e.g., *ACTA2*, *COL1A1*, *FN1*, and *VIM*) at both transcript and protein levels; (3) functional remodeling—demonstrable acquisition of mesenchymal-associated functions (e.g., collagen contraction, enhanced migration, and matrix stiffening) or microenvironmental reprogramming capacity. Single-marker expression or transcriptomic correlation alone is insufficient. Likewise, acute inflammatory activation or *in vitro* culture artifacts should be excluded through appropriate temporal controls and *in vivo* validation.

## The mechanisms of MD

The molecular wiring underlying MD draws upon signaling and epigenetic circuitry that is well characterized across multiple cancer types and fibrotic conditions. Its conservation supports the application of these regulatory modules to the prostate TME. The progression of MD relies on a sophisticated network composed of extracellular signals, intracellular signal transduction, core transcriptional machinery, and epigenetic modifications. These components function in a highly coordinated manner to initiate and sustain MD. The mechanisms underlying MD can therefore be organized as a multilayer regulatory framework rather than as a single linear pathway. Persistent microenvironmental inputs, including profibrotic cytokines, inflammatory signals, mechanical stress, metabolic imbalance, and hypoxia, provide the contextual pressure for cellular plasticity. These inputs activate conserved signaling modules, including TGF-β/SMAD, Wnt/β-catenin, Hippo/YAP-TAZ, NF-κB/STAT3, and PI3K/AKT/mTOR signaling.^24^ Through pathway-linked regulatory mediators, these signaling modules may functionally converge on mesenchymal-associated gene-expression programs characterized by extracellular matrix remodeling, cytoskeletal reorganization, contractility, and migratory capacity. Epigenetic mechanisms may further stabilize or reinforce these transcriptional states.

### Extracellular factors that drive MD

Cells in the TME continuously release various signaling molecules, driving the initiation and maintenance of the MD program. Among immune cells, macrophages are major contributors: factors such as TGF-β and EGF secreted by M2 macrophages directly drive EMT, endowing cancer cells with the ability to migrate.^25^ Beyond macrophages, neutrophils release Gasdermin D-dependent neutrophil extracellular traps (NETs), which act as potent inflammatory signals to directly promote mesenchymal transition.^26^ As the major signal source, cancer cells actively reshape the microenvironment through autocrine and paracrine signaling. They secrete TGF-β to initiate EndoMT and activate CAFs.^27^ Tumor-derived Wnt ligands activate the β-catenin pathway in surrounding cells (e.g., endothelial cells), further promoting EndoMT.^28^

### Intrinsic drivers of MD

MD is coordinately driven by the synergistic interplay of multiple conserved signaling pathways, including TGF-β/SMAD, Hippo/YAP-TAZ, Wnt/β-catenin, NF-κB, PI3K/AKT/mTOR, and histone modification, which collectively orchestrate the formation of a tumor fibrotic matrix and propel malignant progression (Figure 2).

#### TGF-β/SMAD pathway

A central orchestrator is the TGF-β/SMAD pathway. Its core transcriptional effector, Smad3, dominates the cellular reprogramming that drives MMT.^29^ This signaling axis within leukocytes not only promotes the transformation of tumor-associated macrophages into fibroblasts but also inhibits the anti-tumor cytotoxicity of CD8^+^ T cells and NK cells in the TME, thereby facilitating tumor growth.^30^

#### Wnt/β-catenin pathway

The Wnt/β-catenin pathway also plays a critical role, often activated through the TEAD1/BRD4-dependent transcriptional upregulation of ligands like Wnt4, which in turn mediates fibroblast-to-myofibroblast transition.^31^ During EndoMT, a crosstalk between the Wnt/β-catenin and TGF-β/Smad pathways collaboratively induces phenotypic transformation, with molecules, such as S100A4, α-SMA, DKK-3, and β-catenin reported to be involved.^32^^,^^33^

#### PI3K/AKT/mTOR and CX3CR1/MyD88/NF-κB pathway

Additional pathways contribute to MD across different cellular contexts. The PI3K-AKT-mTOR-p70S6K signaling axis is a potent driver of fibroblast mesenchymal transition (FMT) in cardiac valve mesenchymal cells, lung fibroblasts, and hepatic stellate cells.^34^ This cascade drives AMT in breast and pancreatic cancer models and has similarly been shown to induce MMT in a bone marrow infection model.^35^ Moreover, upstream inflammatory signals can epigenetically prime the process. In pulmonary fibrosis, the expression of S100A4 is transcriptionally regulated by the CX3CR1/MyD88-NF-κB signaling axis during monocyte-macrophage differentiation. The upregulated S100A4 protein ultimately endows fibroblasts with the ability to form stress fibers on a rigid matrix, driving their terminal differentiation into myofibroblasts.^36^

### Epigenetic modulation mechanism

To ensure the stability of the MD state, cells also stabilize this new identity through epigenetic mechanisms. DNA methylation silences the expression of the promoter of the tumor suppressor gene RASAL1 by increasing its methylation level, thereby relieving the inhibition of transcriptional repressors such as Snail and Twist.^37^ In AMT, the CpG island of the α-SMA gene undergoes demethylation, causing its expression to be upregulated.^38^ Activating histone modification markers can initiate and enhance the transcription of genes related to the mesenchymal phenotype.^39^ The global histone methylation patterns of H3K4me3, H3K9me2, and H3K27me3, etc. were significantly altered in EndoMT, affecting the transcription of downstream genes.^40^ Histone deacetylase (HDAC) and histone acetyltransferase (HAT) are important to regulate the expression of EndoMT-related genes.^41^ Collectively, these findings indicate that epigenetic mechanisms contribute substantially to MD.

## MD in non-epithelial cells

MD describes the reprogramming process in which various differentiated cells lose their original identities and acquire mesenchymal phenotypes under pathological stimulation.^6^ In PCa, this process is not limited to epithelial cells but involves a wide range of non-epithelial cells in the TME, jointly driving malignant progression. The following will specifically elaborate on several key forms of MD (Table 1).

**Table 1:** Cellular sources, molecular markers and regulatory factors, and potential therapeutic targets of the main types of MD

MD types	Initiating cells	Cell-specific markers^a^	Mesenchymal-related markers^a^	Regulatory factors	Potential therapeutic strategies
EndoMT	endothelial cell	CD31, Collagen Ⅳ, VEGFR, *CDH5*, *VWF*,^42^*CLDN5*,^43^*TEK***,***TJP1*,^44^*OCLN*, *EMCN*,^45^*MCAM*, *CLDN11*^46^	FSP-1, α-SMA,^42^ MMP,^44^*FAP*, *CD44*,^46^*COL1A1*, *COL1A2*, *COL3A1*, *VIM*, *CDH2*,^42^*FN1*^47^	TGF-β, Notch,^44^ Wnt5b, EVs,^48^ ROS, IL-1β, GSK-3β, HSP90α, HSPB1, OPN,^49^ PAI-1,^50^ Jagged1, RBP-Jκ^51^	TGF-β inhibitor (NEO212),^52^ TGFβRI inhibitor (Galunisertib),^44^ NMT inhibitor (5-aza-2′-deoxycytidine), ROCK/FAK inhibitors^53^
PFT	pericyte	NG2, CD146, NESTIN,^54^ PDGFRβ, CD73,^55^*Cthrc1*, *Mfap5*^54^	α-SMA, *S100A4*, *COL1A1*,^19^*ACTA2*, *FN1*, *Nme2*, *Gas5*^54^	PDGF,^56^ TGF-β,^54^ PDGFRβ, Smad2/3, YAP, lncRNA GAS5,^56^ ECM stiffness,^57^ Notch1^58^	PDGFRβ inhibitor (Imatinib, Sunitinib), AKT inhibitor (MK-2206),^59^ SMAD inhibitor (SARA),^54^ CTGF knockdown,^57^ Notch1 inhibitor,^58^ lncRNA GAS5^56^
AMT	adipocyte	*Becn1*,^60^*Fabp4*,^61^*FABP5*, *LPIN1*, *EBF2*, *AGT*,^62^*Adipoq*, *Lep*, *Pparg*, *Plin1*,^7^*Perilipin*^61^	*ACTA2*, *COL1A1*, *FN1*,^60^*SFRP2*, *NCOR2*, *KLF7*, *MFAP4*, *PAI1*, *COL1A2*, *COL5A1*, *TNC*,^62^*CD44*, *ICAM1*, *CD9*, *Ly6a*^7^	TGF-β,^63^ Wnt5a,^62^ YAP/TAZ,^60^ PDGFRα^64^	TGF-inhibitor (Mirabegron),^63^ GPX4 inhibitor (RSL3),^65^ Hippo pathway inhibitor^66^ YAP/TAZ inhibitor (Verteporfin)^60^
MMT	macrophage	CD68, F4/80, CD206,^29^*Csf1r*, *Ccr5*, *H2-eb1*, *H2-ab1*, *Cd74*^29^	α-SMA, FAP, FN1, *S100A4*, *CCN2*, *COL1A1*, *COL1A2*, *POSTN*, *HES1*, *ADAM9*^67^^,^^68^	IFITM3, ITGB3,^69^ TGF-β, Smad3,^29^ RUNX1,^67^ STAT6,^68^ CDH11, CCR8, STAT1, STAT3,^24^ EGFR-YAP,^35^ ALKBH5/IL-11 molecular axis^70^	SMAD3 inhibitor (SIS3), ALK5 inhibitor,^29^ RUNX1 inhibitor (RO5-3335),^67^ ALKBH5 inhibitor,^70^ EGFR inhibitor (Gefitinib, Erlotinib), YAP/TEAD inhibitor (Verteporfin),^35^ STAT6 inhibitor^68^
FMT	fibroblast	*IGFBP6*, *FABP4*, *DCN*^71^	*SULF1*, *COL1A1*, *LRRC15*^71^	TGF-βR, Smad2/3/4/7, MAPK, PI3K/AKT, WNT^72^	ncRNA,^72^ PPARγ agonists (Troglitazone)^73^

Cell lineage and structural markers: Endothelial: CDH5, cadherin 5 (VE-cadherin); CLDN, claudin; EMCN, endomucin; MCAM, melanoma cell adhesion molecule (CD146); OCLN, occludin; TEK, receptor tyrosine kinase (Tie2); TJP1, tight junction protein 1 (ZO-1); VEGFR, vascular endothelial growth factor receptor; VWF, von Willebrand factor. Pericyte: CSPG4 (NG2), chondroitin sulfate proteoglycan 4; NES, nestin; PDGFRβ, platelet-derived growth factor receptor beta. Adipocyte: ADIPOQ, adiponectin; AGT, angiotensinogen; BECN1, beclin 1; EBF2, early B cell factor 2; FABP, fatty acid binding protein; LEP, leptin; LPIN1, lipin 1; PLIN1, perilipin 1; PPARG, peroxisome proliferator-activated receptor gamma. Macrophage: CSF1R, colony stimulating factor 1 receptor; CCR5, C-C motif chemokine receptor 5. Fibroblast: DCN, decorin; IGFBP6, insulin like growth factor binding protein 6; LRRC15, leucine rich repeat containing 15.

Mesenchymal and matrix remodeling markers: α-SMA (ACTA2), alpha-smooth muscle actin; COL1A1, collagen type I alpha 1 chain; FAP, fibroblast activation protein; FN1, fibronectin 1; FSP-1 (S100A4), fibroblast-specific protein 1; MFAP4, microfibril associated protein 4; MMP, matrix metalloproteinase; NCOR2, nuclear receptor corepressor 2; NME2, nucleoside diphosphate kinase 2; POSTN, periostin; SFRP2, secreted frizzled related protein 2; SULF1, sulfatase 1; TNC, tenascin C; VIM, vimentin.

Transcriptional regulators: CTGF (CCN2), connective tissue growth factor; EGFR, epidermal growth factor receptor; FAK, focal adhesion kinase; GSK-3β, glycogen synthase kinase 3 beta; HSP90α, heat shock protein 90 alpha; HSPB1, heat shock protein family B member 1 (HSP27); ITGB3, integrin subunit beta 3; MAPK/ERK, mitogen-activated protein kinase; NOTCH, neurogenic locus notch homolog protein; PDGFR, platelet-derived growth factor receptor; PI3K/AKT, phosphoinositide 3-kinase/protein kinase B; ROCK, Rho-associated protein kinase; ROS, reactive oxygen species; RUNX1, runt related transcription factor 1; SMAD, mothers against decapentaplegic homolog; STAT, signal transducer and activator of transcription. CDH11, cadherin 11; CDH2, cadherin 2 (N-cadherin); CTHRC1, collagen triple helix repeat containing 1; HES1, hes family bHLH transcription factor 1; IFITM3, interferon induced transmembrane protein 3; OPN (SPP1), osteopontin; PAI-1 (SERPINE1), plasminogen activator inhibitor 1; S100A4, S100 calcium binding protein A4; ECM, extracellular matrix; EVs, extracellular vesicles.

Therapeutic strategies and inhibitors: NEO212, a conjugate of temozolomide and perillyl alcohol; MK-2206, an allosteric AKT inhibitor; SARA, smad anchor for receptor activation; GAS5, Growth Arrest-Specific 5; GPX4, glutathione peroxidase 4; RSL3, ras-selective lethal small molecule 3; SIS3, specific inhibitor of smad3.

a Italicized entries in these columns indicate gene symbols.

### MMT

MMT refers to the process by which macrophages transform into myofibroblasts through phenotypic conversion under specific pathological conditions. This transformation is characterized by the co-expression of macrophage markers (e.g., CD68/F4/80) and myofibroblast markers (e.g., α-SMA) and has been identified as a key contributor to fibrotic pathology in multiple organs, including the heart, kidney, lung, and skeletal muscle.^74^ Substantial evidence indicates that macrophage-myofibroblast transformation exists in various diseases. In 2008, a study using immunostaining for α-SMA and CD68 revealed that cells in human placental villi layer also express myofibroblast and macrophage markers, leading to the first proposal of the concept of MMT.^75^ Subsequent studies further confirmed the occurrence of MMT in different pathological scenarios. In the ischemia-reperfusion model, bone marrow-derived macrophages can differentiate into α-SMA^+^ myofibroblasts through cellular senescence.^69^ Furthermore, during ocular surface inflammation, macrophages exhibit the capacity to transdifferentiate into myofibroblasts.^76^ During tumor progression, macrophages in the Lewis lung cancer model express the dual markers of α-SMA and F4/80, confirming that MMT produces a large amount of myCAFs.^67^ In OSCC, studies have systematically revealed the dual roles of MMT in promoting cancer and immunosuppression in OSCC, and the CAFs derived from macrophages promote matrix hardening and immune escape.^8^

Regarding the molecular mechanisms of MMT in tumor environment, TGF-β1/Smad3, as a classic pro-fibrotic signal, drives macrophages to express α-SMA and collagen I in tumors such as non-small cell lung cancer (NSCLC) and OSCC, forming pro-tumoral CAFs.^8^^,^^29^ Runx1 as TGF-β1/Smad3 downstream transcription factor, can regulate MMT gene networks. It has been confirmed as a key regulatory factor in NSCLC.^67^ In addition, the JAK/STAT, STING/TBK, and other pathways are cross-regulated with TGF-β1/Smad3, jointly affecting the polarization state of macrophages and promoting their transformation into myofibroblasts.^68^^,^^77^

Epigenetically, ALKBH5-m6A-IL-11 enhances MMT by regulating the m6A modification of IL-11 mRNA and promotes cardiac fibrosis.^70^ CAFs-derived extracellular vesicles deliver lncRNA SNHG12, which acts as a molecular sponge for miR-181a-5p, thereby de-repressing Smad3 expression and accelerating the MMT of M2 macrophages.^78^ Macrophage-secreted exosomes carry specific microRNAs that activate the MAPK/ERK pathway in fibroblasts and indirectly promote MMT.^79^

### EndoMT

EndoMT is a process in which endothelial cells lose their specific markers (such as CD31, VE-cadherin, and vWF) and acquire mesenchymal phenotypes (such as α-SMA, fibronectin, vimentin, and N-cadherin), along with increased abilities in extracellular matrix synthesis.^42^ In the TME, EndoMT serves as both a driver of structural remodeling, which constructs abnormal blood vessels and dense matrix, and a hub of functional regulation, promoting tumor cell invasion, metastasis, immune escape, and treatment resistance.

Multiple studies have shown that EndoMT promotes its development in different tumors and plays a role at different stages of tumor progression.^48^ The earliest observation of EndoMT came from a 2007 study by Zeisberg et al., using a mouse model of melanoma. This phenomenon was later confirmed in human patients.^27^ In this cohort, EndoMT, evidenced by CD31^+^/α-SMA^+^ endothelial cells, was associated with tumor infiltration.^80^ In the TME, osteopontin (OPN) promotes tumor invasion and metastasis through EndoMT.^49^ In cervical squamous cell carcinoma, CAFs-derived PAI-1 triggers EndoMT in lymphatic endothelial cells, facilitating lymphatic metastasis.^50^ In osteosarcoma, multiplex immunohistochemistry revealed endothelial cells that expressed CD31 and EMCN alongside α-SMA and FSP1, indicating EndoMT.^45^ In glioblastoma, EndoMT occurs in cerebrovascular endothelial cells, contributing to blood-brain barrier disruption and enhanced tumor invasion.^52^ Similarly, studies using 3D microfluidic models of breast cancer further indicate that TGF-β/Notch-mediated EndoMT promotes cancer cell invasiveness and angiogenesis.^44^^,^^81^

The molecular regulation of EndoMT varies considerably across cancer types, and the core signaling network centers primarily on pathways such as TGF-β/Smad, Notch, and Wnt/β-catenin.

The TGF-β/Smad pathway is a major inducer of EndoMT. In the TME, TGF-β and TNF-α induce EndoMT in microvascular endothelial cells, leading to increased vascular permeability and cancer cell extravasation.^47^^,^^82^ TGF-β1 activates Smad2/3/4 and upregulates the transcription factors Snail/Slug, driving endothelial cells to lose endothelial markers (such as CD31 and VE-cadherin) and acquire mesenchymal phenotypes such as α-SMA and fibronectin.^47^

Notch signaling (Notch1, Jagged1, and RBP-Jκ) works in synergy with TGF-β to further enhance the expression of Snail/Slug, promoting vascular remodeling and tumor invasion.^40^^,^^51^ The Wnt/β-catenin pathway (Wnt3a, β-catenin, GSK-3β, and Zeb1/2) induced nuclear translocation of β-catenin activates the ZEB1/2 transcriptional network, cooperatively regulating the migration, proliferation and matrix remodeling of vascular cells.^83^

The inflammatory/cytokine network (IL-1β, TNF-α, IL-6, NF-κB, and STAT3) accelerates EndoMT in the inflammatory microenvironment, which upregulates Snail and Slug through NF-κB/STAT3, forming a positive feedback loop.^84^ Mechanical stress and matrix stiffness (shear stress, YAP/TAZ) amplify TGF-β signaling by activating YAP/TAZ, further promoting the transformation of endothelial cells into mesenchymal phenotypes.^28^^,^^85^ Finally, non-coding RNAs contribute importantly by regulating the aforementioned pathways: the miR-200 family directly targets Snail and inhibits EndoMT; miR-21 promotes EndoMT by inhibiting SMAD7 and activating TGF-β/Smad.^86^ LncRNA-MALAT1 regulates the activity of miRNA through the sponge effect, indirectly affecting the process of EndoMT.^87^

These pathways interweave with each other, forming a complex and cancer-specific regulatory network, which jointly suggests the key role of EndoMT in tumor progression, vascular remodeling and metastasis.

In PCa, EndoMT-related evidence is strongest at the level of hypoxia-associated vascular remodeling and endothelial dysfunction. Hypoxic prostate tumor niches are linked to aggressive phenotypes, therapy resistance, and altered stromal-vascular organization, providing a biologically permissive context for endothelial plasticity.^88^^,^^89^

### PFT

Pericytes are important cells for maintaining microvascular homeostasis. They maintain microvascular homeostasis through expression of PDGFRβ, NG2, CD146, and regulation of vascular stability, local blood flow, and endothelial signaling.^55^^,^^90^ In pathological environments such as tumors, signals like TGF-β can induce phenotypic reprogramming of pericytes, namely PFT. This process is manifested as the downregulation of pericyte markers and the upregulation of myofibroblast markers (e.g., α-SMA and COL1A1).^91^

Hosaka et al. demonstrated that PDGF-BB drives the transformation of perivascular cells into CAFs via PDGFRβ signaling, thereby promoting tumor progression and metastasis.^19^^,^^92^ This observation is further supported by Butti et al., who reported pericyte-to-myofibroblast transdifferentiation across multiple solid tumor types.^93^ Notably, high-dose radiation (e.g., 20 Gy) has been shown to induce pericyte conversion into myofibroblasts, exacerbating vascular dysfunction in tumors.^94^^,^^95^ Single-cell sequencing analyses have identified pericytes as a major cellular origin of metastasis-associated fibroblasts, underscoring the prevalence of PFT.^96^ Collectively, these studies indicate that PFT occurs widely in cancers, such as pancreatic carcinoma, lung cancer, and peritoneal metastasis, where it contributes to TME remodeling through CAFs generation.

The molecular mechanism of PFT involves multi-pathway synergy and cascade activation, and its core driving force stems from the PDGF-BB/PDGFRβ signaling.^19^ PDGF-BB initiates the PFT process by binding to PDGFRβ on the surface of pericytes, activating the downstream MAPK/ERK and PI3K/AKT pathways, inducing pericytes to break away from blood vessels and upregulating myofibroblast markers (such as α-SMA and COL1A1).^93^^,^^97^ The TGF-β/Smad pathway works in synergy with PDGF-BB to promote the transcription of myofibroblast-related genes and the synthesis of extracellular matrix, thereby consolidating the PFT phenotypes.^54^^,^^98^ Radiotherapy or chemotherapy induces phosphorylation of AKT and generates ROS, forming positive feedback to accelerate PFT.^94^ In the TME, the elevated matrix stiffness activates the nuclear translocation of YAP/TAZ through the integrin-FAK pathway, thereby regulating the expression of pro-fibrotic genes and inducing the transformation of pericytes into myofibroblasts.^57^ The Notch/Wnt pathway regulates the transcriptional network in the pericytes-basement membrane interaction, indirectly influencing PDGFR-β activity and participating in the regulation of PFT.^58^ In addition, LncRNA GAS5 indirectly affects the role of the PDGF-BB/PDGFR-β pathway in pulmonary PFT by regulating KDM5B-mediated PDGFRα/β transcriptional inhibition.^56^

In PCa, evidence for PFT is mainly indirect. Perivascular stromal remodeling, PDGFRβ-positive fibroblast-like populations, and ACTA2-positive CAF-enriched niches have been described in prostate tumors, suggesting that vascular-associated mesenchymal cells contribute to matrix remodeling and stromal expansion. In summary, PFT is orchestrated by multiple synergistic signaling pathways, including PDGF-BB/PDGFRβ, TGF-β/Smad, and AKT/ROS. It drives pericytes to adopt a CAFs-like phenotype, conferring robust capabilities in extracellular matrix remodeling, vascular modulation, and immunosuppression.

### AMT

AMT refers to the loss of adipocyte characteristics (such as PPAR-γ, PLIN1, etc.) in mature adipocytes under fibrotic stimulation and their transformation into myofibroblastic phenotypes (α-SMA, COL1A1, FN1).^7^ In the TME, AMT is linked to obesity-driven tumor progression through lipid mobilization and ECM stiffening.

In breast cancer, adipocytes in tumor-invasive mammary fat are induced to dedifferentiate beside tumor cells, forming fibroblast-like precursors and integrating into the tumor matrix to provide ECM, angiogenic factors, and energy matrix, significantly accelerating tumor invasion and metastasis.^65^ In pancreatic cancer, the white adipose tissue (WAT) surrounding the tumor undergoes lipolysis and fibrosis changes when pancreatic cancer invades, releasing free fatty acids and cytokines, which promote the migration of cancer cells.^7^^,^^99^ Furthermore, the review indicates that the fat cells surrounding tumors are generally referred to as “cancer-associated adipocytes” (CAAs). Their AMT process is highly overlapping with EMT and CAFs activation, thereby forming an obesity-driven TME.^100^

AMT has also been observed in other tissue contexts. In patients with systemic sclerosis, the subcutaneous adipose tissue loses the expression of adiponectin and perilipin, and then α-SMA^+^ cells appear, confirming the key role of AMT in skin fibrosis.^101^ In pulmonary fibrosis models, adipocytes undergo AMT and produce collagen.^61^ Adipoq^+^ Pre-bone marrow adipocytes (MALP) in *Staphylococcus aureus* secreted by macrophages trigger EGFR-mTOR-YAP signals, quickly into the α-SMA^+^ myofibroblasts to form fibrous cysts, hindering blood flow, and the penetration of antibiotics.^35^

The transformation of adipocytes into myofibroblasts involves multiple cross-signaling pathways, such as TGF-β, Wnt, PDGFRα, and EGFR-mTOR-YAP.^61^ TGF-β signaling, through the activation of SMAD2/3, which directly induces the expression of pro-fibrotic genes, such as α-SMA, is central to the AMT. Wnt/β-catenin pathway (involving Wnt3a and β-catenin) and TGF-β signal coordination, further promote myofibroblast differentiation.^62^ The PDGFRα-CD9 pathway marks PDGFRα-positive cells with high CD9 expression, and upregulates the expression of collagen and α-SMA after their activation.^61^^,^^64^ The Hippo-YAP/TAZ pathway is activated in response to mechanical tension or the AREG of EGFR signaling. The activated YAP/TAZ, together with the TEAD transcription factor, promotes the expression of myofibroblast-related genes.^35^^,^^66^ Meanwhile, metabolic stress states (such as low PPAR-γ activity, oxidative stress, and hypoxic environment) can prompt mature adipocytes to undergo dedifferentiation and revert to mesenchymal precursor cells. In PCa, hypoxic niches promote aggressive stromal phenotypes and CAF activation,^102^ and the HIF1α-PHD1-FOXA1 axis directly couples hypoxic signaling to FOXA1-dependent luminal identity loss, offering a prostate-specific mechanism linking hypoxia to mesenchymal skewing.^103^ These dedifferentiated cells are subsequently reprogrammed in the fibrotic microenvironment and differentiate into myofibroblasts, exacerbating tissue fibrosis.

### Mesenchymal transition of other cells

Fibroblasts, for example, can be activated via the CD44/αvβ3-AKT/ERK-Twist1 pathway stimulated by breast cancer-derived OPN, differentiating into α-SMA^+^ CAFs.^104^ Single-cell trajectory analysis further indicates that alveolar and outer membrane fibroblast subsets in lung cancer gradually acquire a myofibroblast phenotype accompanied by upregulated expression of cytoskeletal genes (such as COL1A1 and ACTA2).^71^ Similarly, noncoding RNAs have been identified as master regulators of FMT in fibrosis.^72^

Immune cells, although not undergoing canonical mesenchymal transition, can upregulate mesenchymal-associated genes and adopt pro-invasive, matrix-remodeling functions. Endoplasmic reticulum stress pathways are upregulated in tumor-associated neutrophils, along with increased levels of BHLHE40 and LOX-1, suggesting that stress signaling drives their pro-tumor phenotype; clinically, LOX-1/AGR2-positive neutrophil subsets also display high expression of invasion-related genes including Vimentin and MMP-9.^105^^,^^106^ Regulatory T cell-like MT-2 cells respond to tumor-secreted midkine by expressing SDC4, thereby enhancing migratory capacity and tumoral accumulation—a behavior reminiscent of mesenchymal invasion.^107^ In summary, these cells actively contribute to tumor invasion by acquiring mesenchymal-like functions such as matrix regulation and enhanced motility.

### Evidence status in PCa

The MD framework builds upon a body of evidence that varies in depth across transition subtypes and experimental systems. MMT has been rigorously validated through lineage tracing and dual-marker immunostaining in renal fibrosis, OSCC, and NSCLC; these studies establish the mechanistic plausibility of macrophage-to-myofibroblast conversion under pathological conditions. EndoMT is supported by histological detection of CD31^+^/α-SMA^+^ endothelial cells in human prostate tissues, and the dominant regulatory pathways (TGF-β/Smad, Notch, Wnt/β-catenin) are well characterized in melanoma, breast cancer, and glioblastoma models. PFT has been mechanistically established via PDGFRβ lineage tracing in pancreatic, lung, and renal cancers. AMT is best characterized in breast and pancreatic cancers, where adipocyte-to-myofibroblast transitions have been functionally linked to tumor progression. In PCa, the microenvironment harbors mesenchymal-skewed stromal populations—including CD31^+^/α-SMA^+^ endothelial cells, PDGFRβ/ACTA2-positive perivascular fibroblasts, and fibrotic periprostatic adipose tissue—that are consistent with MD. The conserved nature of the underlying signaling and epigenetic drivers provides a strong rationale for investigating these transitions in prostate-specific contexts. Prostate-specific lineage-tracing, spatial transcriptomics, and perturbation studies represent critical next steps to validate and refine the MD framework in PCa.

## Clinical implications of mesenchymal drift

Functionally, MD-associated remodeling may contribute to PCa progression through CAF enrichment, ECM deposition/matrix stiffening, vascular remodeling, and immune suppression or altered immune infiltration.^28^^,^^87^ These structural and immune remodeling processes provide a conceptual basis for understanding the association of MD-related states with adverse clinical behavior and therapy resistance in PCa.

### Correlation with patient outcomes

Numerous studies have confirmed that MD has a strong association with enhanced invasiveness of tumors, enhanced metastasis ability and poor prognosis of patients.^108^ In 2009, the “mesenchymal gene expression signature” (MGES) was identified for the first time in glioma. Glioma cells with high expression of MGES exhibit stronger invasiveness, and short-term survival rate.^21^ Subsequently, clinical data analysis has shown that in various solid tumors such as glioblastoma,^109^ breast cancer,^110^^,^^111^ and colorectal cancer,^112^ the high expression of MGES is an independent risk factor for poor prognosis in patients, and it is clearly associated with a significant decrease in the 5-year overall survival rate of patients.^113^ In MMT, a multicenter cohort analysis of NSCLC showed that a high abundance of CAFs derived from MMT was associated with a significant reduction in overall survival, establishing it as an independent poor prognostic factor.^29^^,^^74^ In EndoMT, studies have found that the proportion of EndoMT cells varies significantly among different tumors and is negatively correlated with the prognosis of patients.^114^ In FMT, FAP^+^ myofibroblasts are significantly enriched in solid tumors, and high levels of FAP^+^ fibroblasts are associated with poor prognosis.^73^^,^^115^ In PCa specifically, DNA methylation at mesenchymal regulatory loci stratifies patients by metastatic risk and androgen deprivation therapy resistance, providing a PCa-specific epigenetic biomarker framework. Therefore, current evidence indicates that the enhancement of MD is associated with increased tumor invasiveness, elevated metastatic potential and reduced survival rate.

### Potential biomarkers and therapeutic targets

Current intervention studies on MD exhibit multi-target characteristics. Studies have shown that small molecule inhibitors targeting the TGF-β pathway, such as Smad3 inhibitors SIS3, ALK5 inhibitors, or macrophage-specific Smad3 knockout, can significantly inhibit MMT and tumor growth.^29^ For RUNX1, as a key regulatory factor of MMT, its inhibitor RO5-3335 or CRISPR-Cas9 targeted knockout can both block the generation of CAFs^67^ (Figure 3).

Regarding PFT, the high expression of PDGF-BB is correlated with the abundance of α-SMA^+^ CAFs and suggests a poor prognosis. PDGFRβ inhibitors imatinib and sunitinib can reduce the number of PFT-derived CAFs and inhibit tumor progression in animal models.^19^ In addition, the combined application of the AKT inhibitor MK-2206 and the antioxidant N-acetylcysteine can prevent chemoradiotherapy induced PFT and enhance therapeutic sensitivity.^59^^,^^116^

In AMT, high levels of AMT markers (such as CD9^+^/PDGFRα^+^, α-SMA^+^ adipocytes) are associated with poorer survival rates in patients with breast cancer and pancreatic cancer and can be used as histological or serum markers for risk stratification.^7^ The β-receptor agonist mirabegron inhibits TGF-β-induced AMT, reduces the expression of Snail and α-SMA, and significantly decreases the number of myofibroblasts in WAT.^63^ The YAP/TAZ inhibitor (verteporfin) can significantly reduce the expression of α-SMA and fibronectin, restore the adipocyte phenotype, and significantly reduce the tumor volume.^60^

During EndoMT, detection of elevated mesenchymal markers such as ANGPT2, FAPα, MMP9, α-SMA, and Vimentin in tumor tissues or blood is helpful for identifying high-risk patients.^53^ The decrease of endothelial markers, such as CD31, VE-cadherin, Claudin-5, and MCAM can reflect vascular dysfunction and poor prognosis.^117^ Treatment for EndoMT can target pathways, such as TGF-β/Smad, Notch, Wnt/β-catenin, and YAP/TAZ. Strategies based on non-coding RNA can be combined with immunotherapy or chemotherapy.^118^ Small molecules or immunotherapies targeting FAPα and MMP9 (such as FAP-CAR-T, MMP9 inhibitors) can act on activated CAFs and disrupt matrix support.^46^^,^^119^

### Challenges and limitations in therapeutic targeting

Despite the growing interest in intercepting MD, several caveats temper the translational optimism surrounding these strategies.

First, the evidentiary basis remains fragmented: most pharmacologic inhibitors discussed above (e.g., TGF-β antagonists, PDGFRβ blockers, YAP/TAZ inhibitors) have been validated primarily in non-PCa models—such as breast, pancreatic, or lung cancer—or in non-malignant fibrosis settings. Their efficacy and safety profiles in PCa remain largely hypothetical and require preclinical validation in PCa-specific systems, including patient-derived organoids and genetically engineered mouse models.

Second, reversibility remains unresolved: while partial reprogramming approaches (e.g., RepSox) can reverse age-related mesenchymal skewing in experimental settings, it is unclear whether therapy-induced MD states in advanced PCa are sufficiently plastic to be pharmacologically reverted, or whether epigenetic fixation has rendered them refractory. Third, broad stromal targeting carries substantial on-target, off-tumor risks: systemic inhibition of TGF-β or CAF-depletion strategies may disrupt physiological wound healing, vascular homeostasis, and immune surveillance, potentially leading to unacceptable toxicities in elderly PCa patients with comorbidities.

Fourth, patient stratification is currently rudimentary: no clinically validated biomarker exists to distinguish MD-high from MD-low tumors in routine practice, raising the risk of treating unselected patient populations. Finally, the temporal dynamics of MD under therapeutic pressure—whether MD accelerates as an early adaptive response to androgen deprivation therapy or emerges as a late resistance mechanism—remain poorly characterized. Addressing these gaps will be essential to move MD-targeted therapy from conceptual promise to clinical reality.

## Unresolved questions and future directions

### Unresolved questions

Several fundamental questions must be addressed to advance the MD framework toward clinical application.

The reversibility of mesenchymal-skewed states remains poorly understood. It is unclear whether these states become epigenetically fixed, such that therapeutic reversal would require direct targeting of chromatin-modifying enzymes, or whether cells retain sufficient plasticity to revert once the driving stimuli are removed. Resolving this distinction has direct implications for whether therapeutic strategies should focus on epigenetic reprogramming or on normalizing the TME. The temporal relationship between MD and disease progression also remains undefined. It is not known whether MD occurs early and contributes to the emergence of resistance to androgen deprivation therapy, or whether it develops later as a consequence of prolonged treatment. Longitudinal studies that track MD-associated changes during disease progression and therapy are needed to establish this timeline.

How prostate-specific factors shape MD trajectories remains poorly understood. These include androgen receptor (AR) signaling, the dense fibromuscular stroma characteristic of the prostate, and the capacity of prostatic fibroblasts to metabolize androgens—all features that are not well represented in the non-prostate models. Addressing this question is essential for determining which aspects of the MD framework can be extrapolated directly from other tumor types and which require prostate-specific investigation.

### Future directions

Testing and refining the MD framework in PCa will require coordinated efforts across multiple fronts. First, genetic lineage-tracing studies in autochthonous PCa models, combined with single-cell and spatial transcriptomics, should be employed to deconvolute CAF origins and quantify the relative contributions of resident fibroblast activation versus *trans*-lineage conversion. Second, patient-derived organoid-stromal co-culture systems offer tractable platforms to test whether MD-associated stromal signatures predict drug sensitivity in a PCa-specific context. Third, standardized multi-omic frameworks that integrate lineage tracing with functional perturbation are essential to establish causal relationships. Finally, integrating PCa-specific datasets that link AR-signaling dynamics, hypoxia-responsive programs, and stromal plasticity will accelerate the translation of the MD framework into clinically actionable insights.

## Conclusion

In summary, MD provides a useful conceptual framework for understanding how stromal, vascular, adipose, and immune compartments may acquire mesenchymal-like features during PCa progression. This process may contribute to extracellular matrix remodeling, immune suppression, vascular dysfunction, and therapy resistance. However, the strength of evidence varies considerably across cell types and experimental systems, and marker co-expression or transcriptomic inference alone should not be interpreted as definitive lineage conversion.

The MD framework offers a testable roadmap for future investigation—one that, with prostate-specific lineage tracing, spatial multi-omics, and perturbation-based validation, can organize emerging data on TME remodeling and guide the development of stromal-targeted biomarkers and therapies in PCa.

## Acknowledgments

This project was supported by the 10.13039/501100001809National Natural Science Foundation of China (82473422), the Taishan Scholar Youth Expert Program of Shandong Province (tsqn202312349), 10.13039/501100007129Natural Science Foundation of Shandong Province (ZR2025QC879), and the Key Talent Program of 10.13039/501100015507Shandong First Medical University.

## Author contributions

Z.Z., writing – review and editing, writing – original draft, visualization, and investigation; F.L., writing – review and editing, conceptualization, visualization, and supervision; S.L., writing – review and editing and methodology; H.N., writing – review and editing, project administration, and supervision; Y.S., study design, editing, supervision, visualization, and funding acquisition; F.W., writing – review and editing, writing – original draft, conceptualization, supervision, and funding acquisition.

## Declaration of interests

All authors declare that they have no known competing financial interests or personal relationships that could have appeared to influence the work reported in this paper.

## Declaration of generative AI and AI-assisted technologies in the writing process

The authors did not use generative AI or AI-assisted technologies to create, modify, or enhance any figures in this manuscript. All schematic figures were redrawn manually by the authors using the online tool BioRender (https://www.biorender.com/).
